# Nécrose digitale du diabétique en hémodialyse

**DOI:** 10.11604/pamj.2014.18.49.4473

**Published:** 2014-05-14

**Authors:** Mohamed El Amrani, Mohammed Benyahia

**Affiliations:** 1 Service de Néphrologie, Dialyse et Transplantation Rénale, Hôpital Militaire d'Instruction Mohammed V, Rabat, Maroc

**Keywords:** Nécrose digitale, diabéte, hémodialyse, digital necrosis, diabetis, hemodialysis

## Image en medicine

La nécrose digitale est une complication invalidante de diversespathologies. Un patient âgé de 60 ans,tabagique chronique et diabétique de type 2 depuis 8 ans compliqué de néphropathie diabétique en hémodialyse, présente depuis un mois des paresthésies et une froideur des mains suivies par l'apparition d'une bulle nécrotique à la pulpe des doigts évoluant rapidement vers la nécrose. L'examen clinique trouve une abolition des pouls radial, cubitalet pédieux ainsi qu'une gangrène sèche de 3 doigts et une ischémie froide des autres doigts sans sclérodactylie (A). L'examen du pied objective une nécrose du 5ème orteil gauche (B). Le doppler artériel montre une athérosclérose diffuse et une abolition du flux dans les artères interdigitales. La radiographie des mains montre des calcifications diffuses dessinant la cartographie artérielle anatomique de la main (C). Le diagnostic de nécrose digitale par athérosclérose calcifiée extensive était retenu. L'athérosclérose avec ses facteurs de risques habituels peut entraîner une artérite digitale du membre supérieur (résultant d'embolies distales à partir de la lésion d'athérosclérose), mais de façon plus exceptionnelle qu'au niveau des membres inférieurs. Les lésions d'ischémies sont aggravées par les calcifications artérielles favorisées par l'urémie chronique et les désordres du métabolisme phosphocalcique connus des patientshémodialysés. Le diagnostic différentiel est posé essentiellement avec les connectivites, les causes hématologiques et les causes toxiques. Le traitement est étiologique mais sutout symptomatique (antalgiques, vasodilatateurs, antibiotiques). L'amputationdoit être différée au maximum, sauf en cas de complication infectieuse, car des lésions qui paraissent irréversibles peuvent guérir par un traitement médical rigoureux.

**Figure 1:**
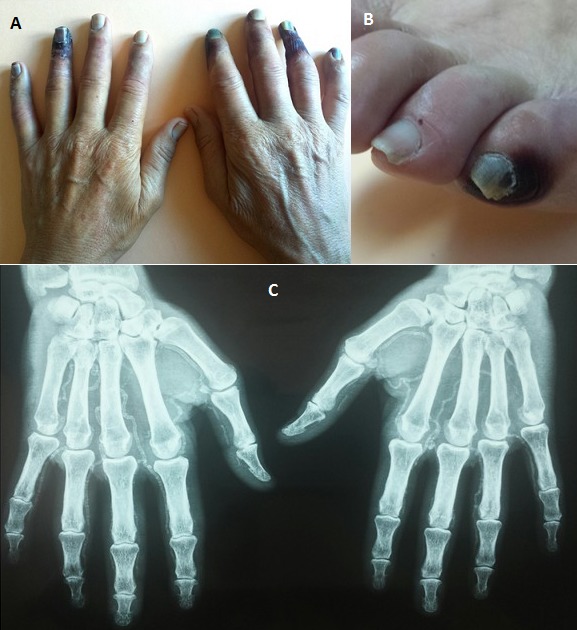
A): Gangrène sèche et ischémie froide des doigts; B): nécrose du 5ème orteil gauche; C): calcifications diffuses du réseau artérielle des mains

